# Proceedings of the Pathological Society of Philadelphia

**Published:** 1859-07

**Authors:** 


### ART. III.—*Proceedings of the Pathological Society of Philadelphia.*

Reported by the SECRETARY.

---

WEDNESDAY EVENING, March 9th, 1859.

The President, DR. LA ROCHE, in the Chair.

DR. ALBERT H. SMITH exhibited several specimens of softening of the brain. One case had during life been attended by pain on motion, but not the slightest feeling was evinced on irritating the skin. A history of the cases was promised for a future meeting.

*Congenital Fissure of the Sternum.*—DR. DA COSTA read the following paper on the case of MR. GROUX, who had presented himself at a former meeting of the Society:—

The recent visit of Mr. Groux to this city enabled me to study some points connected with the action of the heart, which I beg leave to lay before this Society. The subjoined observations were made on five different occasions, some alone, others in the presence and with the aid of several medical friends.

Mr. Groux is the well-known instance of congenital fissure of the sternum. His history has been so often described that I shall not repeat it here.

The fissure is of a V shape, and covered with skin. At its upper portion, near the second rib, and extending downwards, may be seen a distinctly pulsating mass, which has been thought by some to be the auricle, by others the ventricle, by others again the aorta.

The latter view to me seems untenable. During a full expiration a tumor protrudes through the fissure, the upper part of which is clear, or rather somewhat tympanitic on percussion; at the lower part, corresponding to the seat of distinct movement, there is absolute dullness, and a sense of resistance to the percussing finger. Now, if the parts be normal, is it possible for the aorta to be thus displaced?

The correctness of the second opinion, whether it be ventricle or auricle, is more difficult to determine. I believe the pulsating mass to be auricle, and base the opinion—

First. On its peculiar undulatory movement.

Secondly. On its position, which hardly corresponds to the ventricle.

Thirdly. On the difference in the sound heard over it and the part lower down.

Fourthly. On the time of its beat not being the same as that of the apex, between the fifth and sixth ribs.

*Sounds of the Heart.*—Presuming then this to be the auricle, the part below it the right ventricle, the one felt beating above it to be the aorta, I instituted comparative observations on the sounds at these different parts, some with an ordinary stethoscope, others with a small glass stethoscope with a flexible tube, and with this adjusted in a Cammann's stethoscope.

At the *aorta,* with a small glass stethoscope placed in the fissure, two distinct sounds are heard. The first is dull, and more like a continuous vibration; the second, a sharp and accentuated sound. Placing my finger immediately below the stethoscope, two vibrations, or if such an expression be permissible, two sounds, are *felt.* The first and longest vibration corresponds to the first or long sound; the second impulse communicated to the finger is short but well defined, and corresponds to the short, flapping second sound.

If the sounds of the aorta be compared with those of the pulmonary artery, it will be found that the second sound of the former is far more distinct than the second sound of the latter listened to in the first and second intercostal spaces near the fissure. The first sound over the two arteries is much alike. That of the aorta seems, however, longer, and is of lower pitch than that of the pulmonary artery.

This diversity between the sounds over the aorta and pulmonary artery is not owing to the absence of the bone permitting the stethoscope to be applied almost immediately to the aorta. To be sure of this, pieces of India-rubber, and then of bone, were placed in the upper part of the fissure. The character of the sounds remained the same, although those of the aorta were not quite as marked as before. The conclusions from this is evident. It is the sound itself which is different, and it is not its passage through the thoracic walls which determines the usual difference in the sounds perceived over the aorta and pulmonary artery.

Over the left *ventricle,* immediately above the apex, the first sound is dull, but marked, the second is ill defined. There is not any material distinction to be recognized between the sounds of the two ventricles. The first sound of the right ventricle listened to at the lower part of the fissure is clearer and less weighty than that of the left ventricle. The first sound at the apex of the heart and that of the aorta, as heard in the fissure, are much alike, excepting that the first sound of the aorta seems duller, and more like a vibration. This comparison was instituted by using two flexible stethoscopes, each retained at its respective place.

If a small stethoscope be placed over the upper part of the point which is thought to be the right auricle, a sound of very peculiar nature is perceived. This *auricular sound* is not always alike. At times it is similar to a soft, rather musical murmur. At other times it conveys to the ear the idea of liquid, as of the stream of a fountain bursting. Or, again, it may be imitated by filliping with the middle finger. Some of this difference is probably owing to the amount of pressure exerted by the stethoscope. The general character of this auricular sound may be thus expressed: it is a continuous sound, on the whole ringing and high pitched. Although continuous, it is not, however, alike at its commencement and at its end—one is of a lower note than the other, and at its termination a click is heard.

This auricular sound stands in marked contrast to the sounds of the surrounding parts. Below it is heard the dull, lower pitched, booming sound of the ventricles. Above it the aorta sends forth its flapping second sound; not unlike that of the auricle, but lacking the somewhat metallic ring this possesses, and, as far as my comparisons went, not of as high a pitch.

There are many points about this sound of interest, and worthy of careful consideration. It is thought to be an absolute demonstration that the movements of the auricles yield an appreciable sound. I confess, although it is heard exactly over the seat of what may be presumed to be the auricle, that I am by no means fully convinced that it is caused by the movements of the auricle, or of the blood in it. I am not quite sure that it is not a valvular sound produced at the tricuspid orifice, and heard so distinctly, because, owing to the malformation, the stethoscope can be placed directly over, or quite near to, the seat of its production. It has, indeed, very much the flapping character of a valvular sound.

The sharp click with which the sound terminates, seems to me hardly to belong to it. It is heard synchronously with the drawing in of the skin over the part supposed to be the auricle. To test if such a sound might be produced at other parts, by the sudden removal of the small glass stethoscope, DR. J. F. MEIGS, with whom several of the examinations of Mr. Groux were made conjointly, applied a small glass stethoscope with flexible tube over several parts of the chest and neck. A sound precisely similar was produced by its sudden withdrawal from the skin.

*Effects of the Respiration on the Heart.*—The effects of the acts of respiration on the position and sounds of the heart are precisely similar to what I have often observed in other persons. In a full *inspiration* the first sound over the ventricle is lessened, not much over the right, (at the lower portion of the fissure in the sternum,) but very much over the left; the so-called auricular sound disappears entirely. The impulse moves inwards and downwards toward the median line, where it is perceptible to the finger, and also by the distinct motion of pieces of adhesive plaster stuck to the skin. This change of position may be verified by percussion. Near the median line, below the border of the ribs, and below the lower part of the fissure, the previously tympanitic sound becomes dull; but over the seat of previous impulse, between the fifth and sixth ribs, the sound is clear.

In a fixed *expiration* the percussion dullness over the whole heart enlarges. The pulsating mass in the fissure seems to swell. The beat of the heart is felt somewhat higher up, and moves upwards from intercostal space to intercostal space. It is not easy to explain these facts. They are, however, not peculiar to Mr. Groux; I have more than once studied them in lean persons. The probable explanation seems to me to be this: the changed position of the heart in a full expiration causes its beat to be perceived somewhat above its usual seat, and over a larger space. Independently of this, I have been led to conclude from clinical observations, and as the result of vivisections, that during a forced expiration the heart increases in size, and is distended with blood. I have further found that the ventricles are the first to swell. These causes acting together would seem to account for the change of the beat, and its apparent gradual movement upwards.

*Impulse of the Heart.*—With reference to the much-vexed question of the impulse of the heart, whether produced by the systole or diastole, the phenomena presented by Mr. Groux are susceptible of very varied interpretation.

If a flexible stethoscope be placed in the fissure over the aorta, and a finger be applied to the apex beat, it certainly appears, as far as the touch goes, as if the dull first aortic sound corresponded in time to the impulsion against the finger. But the sphygmoscope shows an undeniable difference in time. The blue fluid over the left ventricle, and the red over the aorta, do not rise synchronously. Repeated observations, made by Dr. Meigs and myself, convinced us of this. It seems to me not unreasonable to suppose that the *two* vibrations, which may be felt with the finger, communicate to the fluid a peculiar double movement,—one from the rush of blood, the other from the flapping of the semilunar valves,—which differs from the single motion of the ventricles. This is rendered still more probable by closely watching the columns of the liquid. The one over the ventricle moves straight and with directness; the one placed on the aorta in a wavy, more broken manner. Whether this explanation be adopted or not, the double vibration over the aorta must certainly be taken into account in reasoning on the results obtained by so delicate an instrument as the sphygmoscope, before we pronounce from its indications either in favor of the systolic or diastolic theory.

The question of the impulse did not, however, engage my attention as closely as the points above enumerated. There is so much to be studied in the case of Mr. Groux, and so much care is required in that study, that I preferred to occupy the opportunities I had of examining him in inquiring into some matters that did not appear to me to have been fully described, but which had in connection with other researches engaged my attention. These observations are, of course, only accurate, provided the position of the heart and of the large arteries is the same in Mr. Groux as in other persons. There is no reason to doubt this. Still, in every case of a similar kind, it may be made a question, whether, if Nature has omitted a sternum, she is not likely to have altered the relations of the parts in the cavity of the chest.

---

WEDNESDAY EVENING, March 23, 1859.

Vice-President, DR. STILLÉ, in the Chair.

*Case of Transposition of Viscera, met with in a patient who died of Phthisis.*—Reported by DR. ALBERT H. SMITH.

Joseph Brown, aged thirty, native of England, ostler, entered the Pennsylvania Hospital, on March 3d, 1859. He was emaciated, extremely prostrated, and had marked lividity of the lips and face generally, presenting altogether the appearance of a man almost in articulo mortis. Upon examining him, a loud cavernous respiration, with gurgling, was found at the apex of the left lung, and an absence of respiratory sound at the lower part on that side. Percussion and inspection gave also evidence of an extensive pleuritic effusion, so great as to produce a very marked protuberance posteriorly, and an obliteration of the intercostal spaces. The right lung presented no morbid sign, except a rather rude respiration at the apex. The sounds of the heart were very feeble, but as the patient was so prostrated no very accurate examination was made of the organ, the disease in the lungs being thought sufficient to account for the symptoms. It was, however, remarked that the liver seemed greatly contracted; increased dullness on the left hypochondria was observed, but attracted no particular attention on account of the enormous serous effusion on that side.

Under the use of powerful stimulation he rallied a little; but at no time sufficiently to have warranted a surgical interference for the removal of the fluid in the pleura. On the twelfth of the month he died.

Autopsy.—On opening the thorax the first point of interest was the excessive amount of effusion in the left pleura. There was complete collapse of the left lung, and the whole contents of the thorax were forced over upon the right side of the median line.

On carrying the incision through the abdominal parietes, attention was called to the liver lying upon the left side of the abdomen; a further examination showed that there was a complete transposition of all the thoracic and abdominal viscera, from the innominate artery down to the rectum. The left lung had three lobes, and contained a large cavity in the apex; its pleura was covered with effused and organized lymph. The right lung had two lobes. The heart lay with its apex directed to the right side; the mitral valve and aorta were upon the right, the arch of the latter curving over upon the same side, and sending out the innominate on the left side of the arch. The venæ cavæ were both upon the left side. In the abdominal cavity, on the right side were found the spleen, stomach, descending colon, sigmoid flexure, and rectum; on the left side were the liver, ascending colon, and cæcum. The larger lobe of the liver was upon the left side, though there was much less disparity between the two lobes than usual.

As the examination had to be conducted rapidly, the position of the thoracic duct was overlooked.

DR. HEWSON mentioned a case of similar transposition of the viscera, that had come under his notice. At the autopsy no appreciable cause of death was detected.

*Case of Aneurism of the Aorta near the Diaphragm, rupturing into the pleura.*—DR. ALBERT H. SMITH next exhibited a specimen of aneurism of the aorta.

Archibald Wilkinson, aged twenty-eight, native of Ireland, waiter, entered the Pennsylvania Hospital, March 11, 1859. The history he gave of himself was as follows: About eighteen months since he was in perfect health, having been so for ten years, previous to which he had suffered from an attack of what he called disease of the liver. At the period first mentioned he began to experience loss of appetite, indigestion, occasionally shortness of breath after violent exercise, headache, with a vague sense of uneasiness referred to the epigastrium. These symptoms did not at any time interfere with his occupation, which was not one attended with any great fatigue, until about six months since, when they increased with so much vehemence as to require him to abstain from all exertion. About this time the feeling in the epigastrium became a positive pain, of a sharp, paroxysmal character, much more severe at night. All of the symptoms above mentioned were aggravated; he became emaciated, his appetite left him, he passed sleepless nights, suffered from great mental depression, and was altogether extremely miserable.

When admitted into the Hospital, he was quite feeble, emaciated, having a sallow complexion, and a very haggard, anxious expression of face. He complained of pain in the epigastrium, as mentioned above, and also of occasional pains in the loins and down the legs. He was obstinately constipated. Auscultation and percussion detected no abnormal condition of the lungs or heart. The liver extended considerably below the ribs, and was slightly tender on pressure. In the epigastrium was found, by moderate pressure, a large pulsating tumor, quite apparent to the eye on account of the general emaciation, and both from its producing a prominence and from its pulsation being felt immediately over the vertebral column. On placing the ear over the tumor a very loud murmur could be readily heard, extending for a short distance above the pulsating mass, and downward as far as the bifurcation of the aorta. The urine was highly charged with oxalate of lime crystals.

By means of palliatives his condition was greatly improved; his appetite increased, he slept well, and stated that he felt more comfortable than he had felt for many months. On the morning of the nineteenth, eight days after admission, he was sitting at the head of his bed shaving himself, having used no greater exertion than that of dressing slowly, when he suddenly groaned, rose up from the chair, and fell immediately. He was placed upon the bed, and I saw him about two minutes after. His respiration had then ceased entirely, the pulse at the wrist was not perceptible, though the impulse of the heart could be felt distinctly for more than five minutes after I saw him. Galvanism and powerful stimulation failed to resuscitate him in the least.

The autopsy was made about twelve hours after death. On opening the thorax, both lungs were found completely collapsed, and floating in about a gallon of bloody serum. Raising the right lung, we discovered a firm coagulum of red blood which had formed an exact mould of the pleural cavity, and was so thoroughly coagulated that it could be lifted out entire and weighed; its weight was a little over four pounds and a half. There was no hemorrhage into the peritoneum. The entire thoracic and abdominal viscera were then carefully removed, and the course of the aorta exposed. Just at the position of the diaphragmatic crura was found a sac extending about five inches in length from the lower part of the eleventh dorsal to the upper portion of the third lumbar vertebra, being thus bisected by the diaphragm and constituting a thoracico-abdominal aneurism. On the right side, near the summit of the sac, was an aperture the size of a half dime, through which it had discharged the contents of the aorta into the right pleural cavity. The aorta was then carefully dissected away from the arch to the iliac bifurcation, the diaphragm being detached and removed with it. Over the last dorsal and first two lumbar vertebræ its coats were entirely obliterated, and the bodies of these bones were absorbed to about one-sixth of their extent, the intervertebral substance remaining unaffected. The aneurism was a false, sacculated one; the channel of the aorta continued without change in its calibre except at a point over the first lumbar vertebra, where there was an opening about half an inch in diameter into a sac—which could contain over eight ounces—extending in both directions above and below the orifice of communication. The disease had originated in the inner coat, the external being unimpaired except at the point of rupture, around which the walls were attenuated. The aorta, in its entire course, was studded with patches of atheromatous deposit. The heart was free from any evidence of disease, as also were the lungs. The liver was somewhat enlarged and congested. The colon, from about the middle of the transverse portion down to the sigmoid flexure, was narrowed to a size less than that of the small intestines; there was slight thickening of the coats but no further evidence of disease; above the position at which the diminution of calibre began, the colon was distended to about twice its normal size.

The patient had been, during his whole stay in the Hospital, free from all excitement or exertion; the action of his heart was very tranquil, and the operation he was going through at the time of his death could not, certainly, have increased its force or frequency. The rupture was evidently caused by the contraction of the diaphragm over the middle of the tumor during the act of respiration.

DR. STILLÉ drew attention to the increasing frequency of aneurism. The cause of this is perhaps to be referred to the increasing frequency in the use of alcoholic drinks as suggested by Huss.

*Small Cancer of the Mammary Gland.*—DR. GROSS presented a cancer of the mammary gland, not larger than an English walnut, which he had removed from a patient sixty-four years of age. The disease had existed apparently for about two years. The tumor was hard and dense, but contained under the microscope the elements of cancer. Dr. Gross drew the attention of the Society to the small size of the cancer. He had met with but few instances of such small cancerous tumors of the breast.

DR. WOODWARD stated that several of such cases had come under his observation.

*Calcareous Deposits on the Pleura.*—DR. WOODWARD exhibited a pleura loaded with calcareous deposits, or as it is more usually termed, an ossification of the pleura. The membrane was more than the one-eighth of an inch in thickness, and the deposit of calcareous matter involved its entire substance. A microscopical examination revealed the elements of connective tissue, and numerous granules. On the addition of acetic acid, a free escape of carbonic acid took place.

*Tubercle deposited upon the Mucous Membrane of the Colon and Ileum, in the Mesenteric, Meso-colic and Meso-rectal Glands, and in the Pineal Gland.*—Presented by DR. PACKARD.

T. R. W., aged nearly six years, was attacked with dysenteric symptoms on the twelfth of March. I saw him on the fifteenth. He had been liable to attacks of this kind, or as his mother expressed it, "his bowels had been weak" from early infancy.

The child was always very smart and precocious; had a very large head, light hair, pale blue eyes, and a clear complexion; his mother presents the same physical appearances.

A tendency to stupor which showed itself during the first day or two of my attendance, I ascribed to a mixture containing opium, which had been given him to check his diarrhœa. His pulse ranged from 130 to 150 in the minute; his stomach was irritable to an excessive degree, and his bowels were moved about once in fifteen or twenty minutes. No tympanites existed, nor was there any notable tenderness at any portion of the abdomen. Some gurgling was at times produced by pressure over the right iliac fossa.

The stools were bloody, small, and full of lumps like flesh-washings; there was more pain just before than during their expulsion.

The excessive irritability of the stomach and rectum, which lasted throughout the whole course of the disease, precluded the use of internal remedies. Active counter-irritation to the extremities, and fomentations to the abdomen, were the chief available means.

The symptoms amended somewhat for a day or two; the stools became more natural and rather less frequent, and the slight headache which had troubled the child passed off. No diminution of the irritability of the stomach and rectum could, however, be perceived, and there was a good deal of restlessness and even delirium at night.

At my morning visit on the eighteenth, he seemed a little better in every way; but at noon I was sent for, and found him in a strong convulsion, his surface pale and cold, and his sight gone; pupils dilated and somewhat unequal. By active stimulation and counter-irritation, he was brought out of this state, but sank and died in about three hours.

With the assistance of Dr. Wurts, I made the autopsy, forty-three hours after death: Body very much emaciated. *Head* and *abdomen* only examined.

Skull very large, and well-shaped. Bones thin, and almost entirely without diploë. Dura mater adherent to the calvarium. The brain was large and heavy, and much congested, as were also the arachnoid and pia mater; but no abnormal condition of any portion of it was noted, except the very great softness of the pineal gland. The ventricles contained a very little bloody serum, and about f℥ij escaped from the subarachnoid space. The fifth ventricle was extremely large.

The colon being laid open, its inner surface presented a reddish tinge throughout, owing to the diffusion over it of minute points of vascularity. Its appearance was also somewhat villous, from a soft deposit with which it was covered. Similar conditions were observed in the ileum, and the increased vascularity was especially noticeable in the glands of Peyer.

The lymphatic glands of the mesentery, meso-colon, and meso-rectum, were in a state of great enlargement.

Upon placing under the microscope portions of the pineal gland, of the deposit alluded to as found on the mucous surface of the colon and ileum, and of the mesenteric glands, the peculiar corpuscles of tubercle were at once detected.

Nothing abnormal was observed in any of the other organs.

The hygienic circumstances by which this child had always been surrounded were very favorable; he was well clothed and fed, and in every way well cared for; no disease of any kind seems to have prevailed either in his father's family or his mother's. An older child died several years ago at two years of age, with symptoms which I judge from the account given by the parents to have been analogous to those in the present case; and a younger one is said to be liable to similar attacks of diarrhœa.

The very excessive irritability of the entire alimentary canal, from the outset to the close of the disorder, does not seem to me to be accounted for by the lesions discovered after death, although it would of course have been amply explained had the brain been more extensively diseased.

*Intra-uterine Fracture of the Clavicle.*—DR. KELLER reported the following case.

Mrs. H., of Philadelphia, twenty-seven years of age, became pregnant for the fifth time about the twentieth of December, 1857. Although not robust, she is not of a weakly constitution, and enjoyed, as previously, good health in the first months of her pregnancy.

On riding out in a light carriage, toward the middle of April, she met with an accident. One of the hind-wheels broke off at the axle, and she, leaning forward, fell upon the front wheel so that she injured herself externally on the right side of the abdomen.

She experienced, however, no further disagreeable consequences, and on the first of May felt, for the first time clearly, the motions of the child. In the middle of June she had, without any apparent cause, a severe hemorrhage from the womb, which lasted one day and then ceased entirely.

During the last two months of her pregnancy she felt frequently unwell, and often suffered from pains resembling spasms of the womb. On the twenty-first of September, after several hours of normal pains, she was delivered of a healthy girl. Three days afterwards, the nurse pointed out to me a striking swelling on the neck of the child, which, upon careful examination, I recognized as a completely consolidated fracture of the middle of the clavicle, of which the exterior half stood upward and outward, joined behind in an obtuse angle to the rest of the bone. In an erect position of the body only a very slight deformity was visible, but when the head was much turned it became very apparent.

Considerable discussion followed the report of this case. It was suggested whether the swelling might not have been a morbid growth involving, or springing from the bone.

DR. KELLER could positively affirm that such was not the case. The child was in excellent health, and the deformity so precisely like that resulting from a fractured clavicle that there seemed to him no room for doubt.

DR. PACKARD spoke of the exceeding rarity of such fractures. He could find but one case of intra-uterine fracture of the clavicle reported, that referred to by Malgaigne.

DR. GROSS mentioned the instance placed on record by Glockengiesser, where one hundred and thirty fractures existed in the same fœtus.

*Report of the Committee on the Small-sized Kidney exhibited by* DR. HODGE.—The Committee appointed at the last meeting but one of the Society, to examine the specimen exhibited by Dr. Lenox Hodge, (*see page* 202 *of Proceedings*,) would respectfully report that they placed a portion of the small body, concerning the renal structure of which doubt existed, under the microscope; and that they found in it the tubular character of a normal kidney.

JOHN H. PACKARD,
WILLIAM A. HAMMOND.

---

WEDNESDAY EVENING, April 13th, 1859.

Vice-President, DR. STILLÉ, in the Chair.

DR. HUTCHINSON exhibited a specimen of fracture of the parietal bone with rupture of the middle meningeal artery, accompanied by extensive extravasation of blood upon the surface of the brain.

*Depressed Fracture of Skull.*—DR. LENOX HODGE presented a specimen of depressed fracture of the skull. The man, from whom the specimen was taken, died a few hours after he was brought to the Pennsylvania Hospital, suffering from the effects of a railroad accident.

The base of the skull was fractured, directly across from the petrous portion of one temporal bone to the other; and a depressed fracture of about two inches in length existed in the parietal bones, crossing the sagittal suture, a little above the occipital bone. This specimen shows the tendency of the internal table to exhibit a more extended fracture, and a more marked depression than that indicated by the external table. To test the reality of this difference, it was suggested to me to take a plaster mould of the injury of the external table. I did so; and the difference appeared still more marked, when an elevation was compared with an elevation at the same time, and not an elevated surface with a depressed surface at different times.

The Secretary presented, for DR. JOHN K. KANE, a specimen and the history of a *case of Mitral Disease, accompanied by a musical sound, together with hypertrophy of both ventricles, without dilatation, and perhaps even with contraction of the cavities.*—John O'Brien, a white boy, aged seventeen years, was a native of Philadelphia, and had always resided there. For some months back he had been an inmate of the House of Refuge, from which institution he had been discharged on account of illness. About seven years before he had a severe inflammatory rheumatism; ever since then had been subject to palpitations of the heart, and also to attacks of dropsy.

When I saw him, for the first time, on the fourth of March last, he was complaining of cough, pains in the abdomen, and dysentery. He was lying in bed, very pale, with a hot, dry skin, and had as many as from ten to thirteen stools per diem. The discharges were composed of blood and mucus, with little or no odor. His abdomen was slightly tender on pressure, but not swelled; his face and eyelids, however, were puffy, and his penis and scrotum were highly œdematous. The heart's action appeared tumultuous and irregular, but the pain in the abdomen and extreme weakness were such, that it was not until three days afterwards that I was enabled to make a critical examination into the state of that organ. The abdominal pain and dysenteric discharge ceased almost immediately under the influence of calomel and opium; but they were followed by an enormous effusion of serum, the penis and scrotum becoming tense with fluid; the eyelids more puffed; the legs œdematous; and the abdomen much distended.

On the seventh of March, the patient appearing better and stronger, I availed myself of the opportunity to examine into the condition of his thoracic viscera, and obtained the following results:—On inspection, I found great bulging of the left side, the intercostal spaces being widened, but not bulged. A little below the axilla of the left side there was a distinct depression of about three inches in diameter. The impulse of the heart, described more particularly hereafter, was also observable. Auscultation detected subcrepitant and coarse sibilant râles throughout both lungs. Percussion produced a clear sound over both lungs as low down as the fourth rib; but below this the sound became dull over both. The dullness, however, was more marked, and the resistance to the finger greater, on the left than on the right side. The cough was at times severe. The expectoration was thick, and of a whitish color. Owing to the great amount of effusion it was impossible to define the heart's limits by percussion, except to the right superiorly, where a distinct dullness was perceptible at the third interspace, three-fourths of an inch to the outside of the right border of the sternum. The impulse was tumultuous, strong, and wavy, and caused such a fluctuation in the mass of fluid contained in the abdomen as made it distinctly visible throughout its whole extent. It was most marked, however, at a point three inches below, and a little to the outside of the left nipple, where two distinct impulses could be seen and felt, one slightly above and a little after the other. The rhythm was exceedingly irregular; the average number of beats to the minute was one hundred and fifty-six. On listening over the left side a peculiar blowing sound was heard, a prolonged whiff, winding up a with a clear musical note, a sol. This sound, or rather these sounds, were loudest at the point of strongest visible impulse, leading to the belief that the mitral orifice was the seat of their production; they could be heard, however, with slight loss of clearness at the aortic cartilage, and more faintly over the whole of the heart's extent. They were apparently coincident with the systole, but the heart's action was so tumultuous and irregular, that it was impossible to say, with certainty, that such was the case. It was equally impossible to determine whether the sounds heard at the aortic cartilage were altogether transmitted from the mitral valves, or whether there was not also some aortic disease. I rather inclined to the latter opinion. Careful auscultation over the third left cartilage and ensiform appendix gave no reason to suspect any valvular disease on the right side of the heart. In both places a clear first and second sound could be distinguished, together with a faint transmitted murmur from the left side. There were no arterial or venous murmurs. The pulse at the wrist was so feeble and irregular that I was unable to ascertain whether it coincided with the heart's impulse or not. The patient complained of inability to lie except on his back, and though he never had pain over the region of the heart, he had frequent palpitations, attended by a sense of suffocation. His urine, which was scanty, and of a pale straw color, assumed a thick curdy appearance on being subjected to the action of heat or nitric acid. His gums were swollen, of a pale blue color, and apt to bleed. His tongue was coated at the centre with a yellow fur, the edges being slimy, and of a bluish tint. His appetite was capricious and weak, and he often suffered from nausea. This last was, no doubt, due in part to the quantities of acidulated liquids which he was in the habit of swallowing, and for which his thirst appeared insatiable. My diagnosis was hypertrophy of both ventricles, together with mitral insufficiency and aortic constriction. I thought it probable also that there were old pericardiac adhesions, and there was certainly coexistent Bright's disease of the kidneys.

My friend, DR. DA COSTA, who was kind enough to visit the case with me two days afterwards, was of the same opinion, but suggested that there might also be constriction of the mitral orifice, as well as insufficiency of its valves.

The treatment of this case was necessarily limited, owing to the weakness of the patient, and his tendency to nausea. A variety of diuretics were tried and discarded. Most of them merely sickened him, while those that were free from this objection appeared to lose their efficacy after a few days. I finally fell back on lemon-juice and the simple carbonated water of the shops, which seemed to be quite as effective, and were certainly much more palatable than any others. With regard to sedatives to allay the heart's action, the same difficulty existed,—digitalis and aconite caused emesis before affecting the heart. Belladonna plasters placed over the cardiac region would influence it favorably for a few hours, but after that they lost their influence. As a temporary relief during palpitation, I found a rag wet with chloroform, and applied over the heart, beneficial. Pains in the lower limbs, referable to œdema, were treated by bandaging, and rubbing with a chloroform liniment. Warm baths, simple expectorants to relieve his cough, and nitromuriatic acid as a tonic, with, now and then, a mild cathartic; these made up the general treatment which I adopted. The symptoms varied but little during the first three weeks of my observations; of course, the amount of œdema was less on some days than others, but it was not until the twenty-third of March that I saw any marked change in the patient's general condition. On the morning of this day, when I paid my visit, I found him dull, sleepy, and feverish; his face, which had all along been pale, was flushed, and his eyes glazed. The action of his heart was more than usually violent, and the blowing sound, with its musical termination, remarkably well defined. He slept heavily all that day until about six in the afternoon, when he was taken with a profuse bleeding at the nose, which lasted half an hour, and was arrested with difficulty. He seemed relieved after this, and when I paid my evening visit, at half-past seven o'clock, he expressed himself as feeling more comfortable than he had done for some days past. His skin was pleasantly cool and moist, and his heart beating more tranquilly. He slept well that night, but on the following morning had several attacks of weakness and partial syncope. When I saw him, however, about ten A.M., he appeared better, his pulse was not weaker, and his heart was more tranquil than usual. He said he felt well and hungry. On listening to the heart's sounds, I was struck by the fact that the musical note, which had remained constant, and which had been more than usually marked the day before, had gone entirely. It never returned. The blowing sound, however, remained, and continued not perceptibly altered in pitch until the end of the case. The patient continued better until the third day after this, when he had a recurrence of the attacks of weakness. From that time he grew weaker, and his dropsy steadily increased. On the third of April, his nurse informed me that he had involuntary evacuations from his bowels. On the morning of the fourth of April, I noticed him picking at his bedclothes in a nervous, undecided manner. That evening I was sent for in a hurry. I reached the house at eight o'clock, and found him very weak, gasping for breath, and complaining of dreadful pain in his limbs. It was evident from his expression of countenance that death was coming on. He fell asleep, however, under the influence of anodynes, and continued sleeping until about one A.M., when he awoke, exclaiming that he was dying. At about two A.M., he again fell into a doze, which continued until it lapsed into death, at five o'clock in the morning of the fifth of April.

A post-mortem examination was made five hours after death, in which I was assisted by DR. DA COSTA and one of his students. There was great general emaciation, but owing to the large amount of dropsical effusion, it was not until we cut into the integuments that it became evident. Before being opened the body was much swelled, and the legs discolored. On opening the chest a quantity of serous fluid escaped. Both lungs were much compressed, the left more than the right, and old resistant adhesions were found throughout the whole extent of both pleuræ. The pericardium was so adherent to the pleura of the left lung that it was very difficult to separate them. On removing the heart in its pericardium from the body, the two surfaces of the pericardium were discovered to be universally adherent to each other, and on cutting into the heart itself, both ventricles had the appearance of being *concentrically* hypertrophied. Further examination of the organ was postponed until post-mortem rigors should have time to subside. On the examination being continued, sixty hours after death, we found the wall of the right ventricle to be as much as one and one-third inches in thickness, its columnæ carneæ being enormously developed. On measuring the transverse diameter of the cavity of the right ventricle at its widest portion, we found the distance from wall to septum to be one and one-third inches. The walls of the left ventricle were not so extensively hypertrophied as those of the right. They were much more so in the neighborhood of the mitral orifice than elsewhere. A measurement taken just below this point gave as much as three-fourths of an inch in thickness, while lower down they measured not quite half an inch. The columnæ carneæ, too, though larger than normal, bore no proportion to those of the right ventricle. The greatest transverse diameter of the left ventricle from wall to septum was one and three-quarter inches. The pulmonary and tricuspid valves and orifices we found to be normal. On examining the mitral opening we found the orifice itself constricted, and the valves thickened and insufficient. The aortic orifice was constricted, and its valves, though insufficient to close it, were considerably thickened. Both auricles were apparently normal, though clots of considerable size were found in both; the right being almost entirely filled by a non-adherent clot, while the left contained a large adherent clot of elongated shape which had its origin directly at the mitral orifice.

The symptoms in this case have been detailed.

The post-mortem examination having verified the diagnosis, it might seem a work of supererogation to designate the particular indications which led to the different diagnostic conclusions. I may venture, however, to invite attention to two points, which seem to me to be worthy of more than a merely passing notice. The first, the fact of two impulses being visible at the point of apex beat taken in connection with the extensive agglutination of the pericardial surfaces. There were other reasons which led me to suspect the existence of pericardial adhesions. The fact of the præcordial interspaces not being bulged, while there was evidently so great an amount of effusion into the cavity of the thorax. The extreme lowness of the point of apex beat, notwithstanding the great abdominal distention, and the knowledge of the previous attack of inflammatory rheumatism already referred to, followed by palpitations, all looked in that direction; the strong double impulse settled the point to a certainty. The second point, for which I would ask consideration, is the musical sound which was produced at the mitral orifice, and which so suddenly disappeared. The action of the heart was so tumultuous that it was impossible to say whether it was coincident with the systole or diastole of the ventricles, and yet its existence caused Dr. Da Costa to suspect constriction of the mitral orifice, as well as disease of the valves. The reason he gave was, that in all the cases of purely cardiac musical sounds which had come under his notice, constriction had been present. The post-mortem results showed this actually to be the case. I am, therefore, constrained to believe that the musical note, which was of very short duration, was not a mere termination of the regurgitant blowing sound, but rather a distinct sound caused by the blood passing through the constricted orifice, and coincident with the diastole of the ventricles. What was its mechanism, and why it ceased, it would be difficult to say. Perhaps a shred of vibratile lymph was its cause, and this being suddenly swept away, or gradually thickened by fresh deposits, the sound was no longer produced. Another hypothesis equally plausible and equally insusceptible of proof, is, that the sound was produced by the resisting edges of the constricted orifice vibrating in the current of the blood, and that these, for some reason, (increased thickening, for instance,) lost their musical resonance. Perhaps the adherent clot found after death in the left auricle, was the cause of its cessation, either by altering the direction of the current, or otherwise.

The heart was referred, for further examination, to a committee.

---

WEDNESDAY EVENING, April 27th, 1859.

Vice-President, DR. STILLÉ, in the Chair.

*Pericarditis, and alteration of the Valves of the Heart.*—DR. HALL exhibited a specimen of Pericarditis, accompanied by changes in the valves, removed by him from a patient of Dr. Martin; the man was about fifty years of age, and had suffered for years from rheumatism.

The symptoms were those usual to the disease. The physical signs were as follows:—

Dullness over the region of the heart, the limits much increased, and the sound flat. Friction sound, with a thrill to the hand or ear, below the left nipple. A murmur during both systole and diastole, was heard loudly over the heart and arteries, and moderately over nearly the whole anterior part of the thorax and in the carotids. The normal sounds were both absent or entirely obscured by the murmur. This was heard most clearly over the aortic valves, and from there it was transmitted down toward the apex.

Post-mortem examination.—The head was not examined. There was a large effusion into the right pleural cavity, with bands of highly organized lymph lining the pleura. The lungs were highly congested at their base, but were otherwise healthy. The pericardium was distended, with about five ounces of amber-colored serum; there were one or two small patches of lymph upon the pericardial surface of the heart. Measurements taken on the spot showed the heart to be affected with eccentric hypertrophy, being enlarged in both diameters. The aortic and mitral valves were thicker than natural, but no decided disease appeared—not so much as was expected. A very small patch of atheromatous deposit was found on one of the curtains of the aortic valves. The right side of the heart was healthy. There was no disease of the kidneys.

The blowing sounds over the aortic valves were, Dr. Hall thought, to be accounted for by the presence of considerable dilatation of the ascending aorta, producing an inaccurate closure of the valves. There was not sufficient change in the structure of the valves to have originated a murmur. Nor was it caused by impoverished blood, since the man did not look in the least degree anæmic.

*Multilocular Ovarian Cyst.*—Presented by DR. HUTCHINSON.

The patient had been tapped during life and an enormous quantity of a tenacious albuminous fluid discharged. The cyst filled the whole abdomen. It sprang from one ovary; the other was healthy, but perhaps somewhat harder than natural.

*Report of the Committee on the Heart, presented by* DR. KANE.—The committee appointed to examine the specimen of supposed concentric hypertrophy of the heart, presented to the Society at its last meeting by Dr. John K. Kane, respectfully report:—

That owing to the fact that the specimen above mentioned had, when submitted to examination, been a lengthened period in alcohol, and was, moreover, greatly mutilated by previous examinations, the committee hesitate to express a decided opinion from personal inspection and measurement. Taking, however, into consideration the careful measurements made by Dr. Kane and recorded in the paper accompanying the specimen in question, and comparing them with numerous measurements made by other observers of normal adult hearts, there does not seem scarcely a doubt of the existence of concentric hypertrophy in the case under consideration.

The committee would refer to the following table of M. Bizot,* showing the capacity of the human heart:—

LEFT VENTRICLE—MALE.

| AGE. | LENGTH. | BREADTH. |
|---|---|---|
| 1 to 4 years . . . . . . | 20 lines. | 31 lines. |
| 50 to 79 " . . . . . | 36 " | $56\frac{2}{3}$ " |
| Average, from 15 to 79 years . . | $34\frac{24}{61}$ " | $54\frac{9}{61}$ " |

LEFT VENTRICLE—FEMALE.

| | | |
|---|---|---|
| 1 to 4 years . . . . . . | $18\frac{1}{2}$ lines. | $29\frac{5}{8}$ lines. |
| 50 to 79 " . . . . . | 31 " | $49\frac{1}{2}$ " |
| Average, from 15 to 89 years . . | $31\frac{1}{30}$ " | $48\frac{23}{30}$ " |

* Cyclopedia of Anatomy and Physiology, art. *Heart*, vol. ii. p. 586.

RIGHT VENTRICLE—MALE.

| | | |
|---|---|---|
| 1 to 4 years . . . . . . | $20\frac{1}{5}$ lines. | $47\frac{4}{5}$ lines. |
| 50 to 79 " . . . . . | $37\frac{1}{3}$ " | 87 " |
| Average, from 15 to 79 years . . | $57\frac{14}{61}$ " | $82\frac{11}{66}$ " |

RIGHT VENTRICLE—FEMALE.

| | | |
|---|---|---|
| 1 to 4 years . . . . . . | $18\frac{7}{8}$ lines. | $44\frac{1}{8}$ lines. |
| 50 to 79 " . . . . . | $35\frac{11}{19}$ " | 76 " |
| Average, from 15 to 89 years . . | 34 " | $76\frac{3}{8}$ " |

WILLIAM A. HAMMOND,
ROBERT P. HARRIS.

---

WEDNESDAY EVENING, May 11, 1859.

The President, DR. LA ROCHE, in the Chair.

*Gunshot Wound of Neck.*—Presented by DR. LENOX HODGE.

J. McC., about thirty years of age, was brought to the Pennsylvania Hospital on the afternoon of Friday, April 29. He had a gunshot wound of the neck. The wound of entrance was on the middle line in front, and over the lower part of the larynx. There was no wound of exit. Respiration had already ceased, but his heart was still beating, the pulse being full and slow, about thirty in a minute. The surface was pallid. A small clot of blood lay on his chest.

Artificial respiration appeared to produce no effect. Thinking that there might be some impediment to the passage of air through the larynx, I enlarged the wound downward, and inserted a tracheotomy-tube. Through this we could readily force the air, but still failed to restore the respiration.

Upon making the post-mortem examination we found that the ball having fractured the cricoid and thyroid cartilages, had passed directly through the larynx and œsophagus, and struck the body of the sixth cervical vertebra; then glancing a little to the right it had torn away a portion of the bone, passed between the transverse processes of the sixth and seventh cervical vertebræ, and lay imbedded in the muscles on the back of the neck. Upon opening the larynx, we saw that in addition to the fractures the mucous membrane was much torn from its attachments, and fell like a valve; and that several small coagula of blood were present. The trachea and bronchia contained no coagula, but a little fluid blood. The membranes of the spinal cord were deeply congested where the ball passed near them, but there seemed to be no solution of continuity. In examining the other organs, we saw nothing worthy of note in this connection.

The point of special interest in the case is, to determine the exact cause of death; was it the injury to the larynx or to the spinal marrow, or both, conjointly, or perhaps still other causes which occasioned it?

DR. HAMMOND stated that a case had come under his notice in New Mexico, where a slug fired from a smooth-bored gun in the hands of an Indian, at a distance of about two hundred yards, split upon the anterior portion of the thyroid cartilage, into two pieces of unequal size, which passed under the skin and made two orifices of exit. One of these was a little posterior to the left ear; the other on the right side, about an inch from the cervical vertebræ. The exact shape of the slug could not be determined, but as Indians are in the habit of chewing their slugs into shape, he judged from those he had seen which had been thus formed that it was an irregular ovoid, three-quarters of an inch in the long, by half an inch in the short diameter. Dr. Hammond further stated that Indians shoot with small charges of powder, which might tend somewhat to account for the fact of the ball not having entered the cartilage. He also alluded to a case reported to him by another medical officer of the army, in which a musket ball had split upon the nasal bones, the pieces entering the eyes.

DR. LA ROCHE made the following communication:—

The medical profession of this city have very lately sustained a severe loss by the death of Dr. Henry Bond, who for about forty years enjoyed among us the well-merited reputation of a judicious practitioner and honorable and high-minded gentleman. He died on the fourth of May last, in the seventieth year of his age.

As Dr. Bond was not a member of this Society, I should not have called attention to the circumstance of his death, had not the disease under which he labored and which finally carried him off, and particularly the morbid appearances discovered on inspection of his body, presented some points of interest.

For some twelve or fifteen years prior to his death, he labored under the usual symptoms of hypertrophy and valvular disease of the heart. About eight or ten months ago, while walking in the Washington Square, he fell to the ground under an attack of apoplexy, and was thence carried to his house, where, under the use of judicious remedies prescribed by Drs. Bell and Hoyt, who in my absence from home were summoned to his assistance, he soon recovered his consciousness. The consequence of the attack was paralysis—almost complete—of the whole left side of the body, as also the organ of speech. From this condition—in which I found him at my first visit with Dr. Bell, who continued to attend him—he in a great measure and rather rapidly recovered; sufficiently so, indeed, to be able to raise his hand and arm with a certain degree of facility, and to walk in and out of doors without assistance, and with little more than a moderate drag of the affected foot. The muscles of his face presented slight evidence of the nature of the disease. His articulation was somewhat thick, slow, and indistinct; the pitch of his voice low, and the force of the sound so feeble as to require the closest attention on the part of those near him to enable him to be understood. His mind appeared clear, though giving evidence from day to day of a tendency to a gradual loss of strength.

On election day he walked out alone to cast his vote, and passed the day as usual, without indicating any sign of increasing disease. He played a game of backgammon with a friend in the evening, remained in conversation with the family until after ten o'clock, when he retired to bed. The next morning, between six and seven o'clock, he was found by his servant, dead on the floor of his chamber. He was lying on his left side, near the bed. His extremities were cold, but relaxed. On the forehead, just over the left eye, was a cut and contusion, surrounded by blood, evidently produced by his striking, in falling, the edge of a bureau situated near the bed. The clothes on the latter were scarcely deranged.

The autopsy was made thirty hours after death, by Dr. Brinton, in presence of Drs. Bell, Wood, Coates, Condie, C. P. La Roche, and myself, and revealed the following appearances :—Occipital protuberance uncommonly prominent, with corresponding thickness of bone. The entire surface of the brain exhibited great venous congestion. The arachnoid membrane, especially toward the summit of the brain, was decidedly opake, with effusion beneath it of a serous fluid, and in patches, of a semi-gelatinous matter. The vessels at the base of the brain were all more or less ossified. In cutting into the substance of the brain, the medullary matter became immediately covered with innumerable small spots of red blood. The choroid plexus was considerably congested. A large amount of serous effusion—probably two ounces—was contained in the lateral and other ventricles. The hippocampus major of the right side was unusually large, infiltrated with fluid, and decidedly softened. The velum interpositum was strongly injected with blood. The substance of the medullary portion of the anterior lobe of the right side of the cerebrum was considerably softened and injected with a pus-like fluid, the softening extending as far back as the descending cornua. The cerebrum was large in size and firm in texture; the convolutions were uncommonly deep. The cerebellum was, compared with the cerebrum, uncommonly small in size; it was congested, but less so than the cerebrum. The cartilages of the ribs were ossified. Old adhesions of the left lung to the costal pleura were discovered. Considerable effusion into the cavity of the pericardium existed. The right auricle contained a very large amount of fluid blood.

The heart was much above the medium size, and on its surface were to be seen large deposits of fatty matter. The coats of the aorta exhibited ossific deposits. The left ventricle was considerably hypertrophied and thickened; the right nearly natural. The semilunar and mitral valves were, especially at the base, thickened and ossified.

The changes found in the heart confirmed fully the opinion which had long been expressed of the nature of the disease of that organ under which Dr. Bond labored so many years. Those found in the brain were sufficient to account for the attack of apoplexy and paralysis. In consequence of not finding in the latter organ any marks of recent disease of a kind calculated to explain the sudden death of the patient, and in view of the nature of the morbid changes in the heart and of the unusual accumulation of blood found in the right auricle and surrounding vessels, several of those present at the autopsy were inclined to look to that organ for the immediate cause of death.

---

WEDNESDAY EVENING, May 25th, 1859.

Vice-President, DR. STILLÉ, in the Chair.

*Four Cases of Wounds of the Liver.*—Presented by DR. GEORGE C. HARLAN.

CASE I.—The specimens on the table were taken from a man killed yesterday afternoon by a fall. He fell from a height of three stories from a ladder. At the time of his admission into the Hospital he was entirely insensible, the respiration was labored, the pulse frequent and full, the pupils contracted, and blood was flowing freely from both ears. There was no reaction, and he died in about half an hour.

A post-mortem examination revealed an extensive fracture of the base of the skull, involving the occiput on both sides of the foramen magnum and the petrous portion of both temporal bones. On the left side of the occiput the separation was wide enough to allow the blade of a large scalpel to be readily introduced into the fissure. There was some blood at the base of the brain, but no clot. The blood had continued to flow from the ear for some time after death. There were lacerations of the cerebellum corresponding in position and extent to the fissures in the occiput. The liver was also injured. There was a laceration on the upper surface of the right lobe, about four inches in length and one-eighth of an inch in depth, presenting an irregular, granular appearance. There were two or three ounces of effused blood in the peritoneal cavity, but no external marks on the abdomen. This is the fourth case of rupture of the liver that has occurred in the Pennsylvania Hospital within the last two months, and as illustrating the frequency of this injury in hospital practice, it may be worth while to give a brief account of the three others.

CASE II.—A few weeks ago an old man was admitted into the Hospital, with injuries inflicted by a railroad accident. The accident occurred at Paoli, twenty miles distant, and when he reached the Hospital he was very much exhausted, and almost pulseless. Though terribly injured he was perfectly conscious, and complained most of dyspnœa and pain in the abdomen. He died in about an hour. Several ribs were found to be broken, the right lung was torn, and there were compound fractures of the leg and arm. There was upwards of a pint of effused blood in the peritoneal cavity. The liver was ruptured in more than twenty places. The lacerations were from an inch to four or five inches in length, and their greatest depth was about a quarter of an inch. They had occurred principally on the right lobe, and occupied both the upper and under surfaces. No signs of external injury of the abdomen were detected.

Of the other two cases, one occurred in Dr. Hutchinson's ward, and he has furnished me with the following notes:—

CASE III.—A man was brought to the Pennsylvania Hospital during the last week of March, having met with serious injuries in consequence of his wagon and a train of cars having come into collision. The man was evidently dying at the time of his admission, about an hour after the accident had occurred, and no very thorough examination was attempted. Death took place in less than three-quarters of an hour.

An autopsy made thirteen hours afterwards revealed an extensive fracture of the skull, also of the three lower true ribs of the left side. The peritoneal cavity contained about four ounces of blood. Upon the upper surface of the left lobe of the liver, at its extreme border, was a stellated rupture, about three-quarters of an inch at the deepest part. The diaphragm was not ruptured; nor was there an external injury in the region of the liver.

For the notes of the fourth case I am indebted to Dr. Hodge:—

CASE IV.—Bernard Bradley, aged twenty-one, was run over by a cart. He had, when first seen, a fractured humerus, and stated that the wheel had also passed over his body. He complained of pain across the abdomen, but had no mark of contusion; no crepitus of the ribs was found. He was very drunk, and had walked in with the assistance of police officers. He was admitted between three and four o'clock A.M.; vomited frequently during the morning; sank suddenly, and died about half-past three o'clock P.M.

Post-mortem examination.—Brain showed nothing worthy of notice. In the cavity of the peritoneum were several ounces of bloody serum; in the pelvis handfuls of clots. The liver was lacerated and contused on its under surface near the gall-bladder.

The experience of the Hospital decidedly confirms the opinion that the liver, next to the brain, is the viscus most likely to suffer from falls and contusions; a fact that its size and weight will readily account for. The prognosis is, of course, always very unfavorable. Where the injury is extensive, it will most probably be followed by fatal depression, on account of the sympathy existing between the abdominal viscera and the other parts of the system by means of the great sympathetic. The three sources of hemorrhage—the hepatic artery, hepatic vein, and vena porta—increase the danger of death before reaction. If the patient should be fortunate enough to pass that stage, he is still subjected to the risk of violent peritonitis, and must overcome the difficulties that the secretion of a gland always presents to the healing of a wound in its tissue. These considerations make recovery appear almost like a miracle, and have induced some surgeons to believe that none but the most superficial wounds of the liver ever heal, and that others are necessarily and invariably fatal.

This is certainly true as a general rule, but numerous decided exceptions are recorded by various authors.

The subject appears, at different times, to have excited a good deal of attention, and experiments have been made on the lower animals. Among others, Dr. Monro, of Edinburgh, removed portions of the liver from rabbits, without permanently injuring their health. The wound cicatrized, as in other parts of the body. Guthrie, in his Commentaries, speaks of three cases, terminating favorably, in which protruded portions of the human liver were excised. Another is mentioned, in Thompson's work on the Liver and Spleen, in which more than three ounces of the organ were removed, and the patient got well. Dr. Smith, of Maryland, reported, in the North American Archives, (vol. i. page 385,) the case of a man, whose liver was wounded by a dagger, made by grinding the point on a common table-knife. Unpleasant symptoms subsided on the fourth day, and the man recovered. Hennen, in his Military Surgery, records a case in which a musket-ball entered between the eighth and ninth ribs behind, two and a half inches from the spine, and passed out in front, between the seventh and eighth ribs, four and a half inches from the sternum. Hemorrhage from both wounds lasted three days, and the posterior continued to pour out a bilious discharge for nearly six weeks. In three months from the time of the accident the patient was in almost perfect health.

The recovery of two other cases is recorded, in which bile was discharged from the wound, in one of which a musket-ball could be felt buried five inches deep in the organ. The man lived several years, though his health was bad, and purulent and bilious matter was constantly discharged from the wound. Dr. Thompson refers, in the work above quoted, to a case of gunshot wound of the liver, in which the patient died some time afterwards of a different disease, and it was found that the ball had passed through the left lobe, about three inches from its lower border.

In nearly all the above cases the injury was followed by jaundice, and in several the characteristic pain in the shoulder was present. How soon the jaundice appeared is not mentioned.

When the gall-bladder is wounded, death is almost inevitable from the constant flow of bile into the peritoneal cavity. Thompson mentions one authentic case of recovery. The patient died of thoracic inflammation two years afterwards, and a bullet was found in the gall-bladder.

In all the cases referred to above, there were external openings. As lacerations from concussion and contusion can only be certainly recognized by examination after death, there is an entire want of statistics in reference to the possibility of their recovery. The want of an exit for the discharge is an unfavorable circumstance, and as they are hardly likely to heal by first intention, their only chance is probably in the formation of an abscess and of peritoneal adhesions.

It may, however, perhaps be possible for recovery to take place even without these adhesions. Dr. Nicol, an English army surgeon, located for some years in India, found, in many instances, cicatrices on the surfaces of the liver, which, in his opinion, must have occurred from the bursting of abscesses into the cavity of the abdomen. Hennen has also seen several similar cases.

Several members of the Seciety alluded to cases in which they had had positive evidence, or thought it exceedingly likely that the liver had been wounded and the wound cicatrized.

*Rapidly Recurring Encephaloid Cancer of the Left Breast.*—Dr. S. W. GROSS presented a specimen of cancer of the breast, from an unmarried servant woman, forty years of age, whose menstrual functions were performed regularly.

It was removed yesterday morning by Professor Gross, and was the fourth operation which has been performed for the same disease in the last twenty months. In the first three operations the gland was but partially removed, leaving the greater portion as the nidus for the new formations. About two years since the first tumor made its appearance, and was extirpated at the expiration of four months. The second soon began to develop itself, and was removed in nine months. In a few weeks the third tumor commenced to grow, and was excised in seven months, and the specimen on the table was removed four months and a half after the last operation.

All of these tumors were tuberculated, firm, and compressible, and inclosed in an imperfect cyst, formed by the condensation of the surrounding cellular tissue. There was no retraction of the nipple, and no involvement of the neighboring lymphatic glands. The subcutaneous veins were enlarged, and the tumor was the seat of sharp, shooting pains, extending to the shoulder.

The woman's general health has always been excellent; she has always made good recoveries from the operations, and there is no cancerous disease in her family.

Physical examination showed that the tumor was encephaloid, which was confirmed by the microscope, a great number of very large cancer-cells being present.

The points of interest in this case are, the rapid recurrence of the cancer; the non-involvement of the lymphatic glands; the absence of the cancerous cachexia; the good health of the patient; and the non-appearance of the tumors at the cicatrices of the former operations.

DR. WOODWARD said that he was much interested in the case which had just been detailed to the Society, and had especially noted the fact of the absence of the symptoms of the cancerous cachexia in this rapidly recurring cancer. The so-called cancerous cachexia is often absent, both in cases of scirrhus, and encephaloid. Cancers may be observed, from time to time, possessing the attributes of malignancy in a terrible degree, running a most rapid course and speedily terminating fatally, but in which none of the so-called cachectic appearances occur at any time. As a general rule, when cachexia exists, there is more or less disease in important internal organs, cancerous infiltration, fatty degeneration, and the like.

On page 201 of the Proceedings, his remarks would seem to indicate that he attributed the cancerous cachexia exclusively to fatty degeneration of the liver. He desired in this place to correct any erroneous impression which might arise from the perusal of that paragraph, which only in part represented his views.

Undoubtedly disease of the liver plays an important part in *some cases* in the production of the appearances called cachectic; we are not yet in a condition to say that it does in all. Nor can we overlook the part which the diseased state of other internal organs, as the mesenteric and other lymphatic glands, the intestinal canal, the lungs, etc. may play in creating a cachexia.

It is necessary to observe the utmost care in such investigations as the present, lest a generalization, based upon a limited number of facts, be overturned on a wider survey. But we are placed in this position: the cancerous cachexia so-called is not constant; cancer may exist without it, and that in any degree of severity; nor are these rare exceptional cases; they are exceedingly numerous, every surgeon can recall them from his own personal observation; and hence is inevitably and logically drawn the inference, that the cachexia is not *necessarily* the companion of cancer; that it is an *accident*, and not an *essential;* and if an accident, we are driven to look beyond the carcinomatous disease to the complications and to the disturbed functions of involved organs for its cause.

If these views be correct, it legitimately results that the diagnostic value of the cachexia is very small. The fact of its absence will not prevent the surgeon from diagnosing cancer in many cases. But it has been said that *when present* the cachexia invariably indicates cancer. This assertion at once starts the question, is the cachexia so peculiar that it can unhesitatingly be called cancerous, *apart* from the evidences of a local cancer? It is to be regretted that this question must be answered in the negative. There is no one symptom belonging to this cachexia that may not be met in other diseases; and in patients free from cancer, but suffering from various chronic diseases, and especially from painful diseases of internal organs, a train of symptoms occasionally arises so exactly imitating the so-called cachexia as to lead to errors in diagnosis even by experienced physicians and surgeons.

The question of cancerous cachexia, and its cause, or causes, was further discussed by several members of the Society.

DR. DARRACH stated that he had had several times occasion to notice that the cancerous cachexia is well pronounced when the lymphatic glands are the seat of the disease.

DR. PACKARD remarked that from some cases which had come under his observation, the cachexia accompanying cancer seemed dependent, in part at least, on the degree of influence directly exerted by the local disease upon the function of blood-formation.

Thus in several cases of small cancerous deposits stricturing the œsophagus or pylorus, and in this way interfering essentially with the supply of alimentary material, the cachexia was very marked. In several others, the rectum being the seat of disease, this symptom was but slightly developed. When, however, extensive formations of cancer take place in the rectum, and especially when they increase rapidly,—when a drain is thus established upon the blood already formed,—the cachexia is apt to be well-pronounced.

Small or slowly-growing cancers, so seated as to interfere with the function of blood-formation, as for instance in the stomach, liver, or lungs, seem, therefore, to be attended with a more marked cachexia than those seated elsewhere. But if the latter are very large, and abundantly nourished, they have a like effect, perhaps, however, owing in great measure to their drafts upon the whole mass of blood. A remark made a few minutes since by Dr. Darrach, that the cachexia is generally well-pronounced when the lymphatic glands are the seat of the disease, seems to afford obvious support to the idea suggested.

Dr. Packard would of course by no means assert that a mere deficiency of blood-material, or a mere drain upon the fully-formed blood, is by itself assignable as the cause of the cachexia in cancer. Whether there is added to either a specific element, and in what that specific element may consist, are questions not yet answered; meanwhile the circumstances mentioned have perhaps some weight, if they are in accordance with general observation.

